# Anaphase-promoting complex/cyclosome protein Cdc27 is a target for curcumin-induced cell cycle arrest and apoptosis

**DOI:** 10.1186/1471-2407-12-44

**Published:** 2012-01-26

**Authors:** Seung Joon Lee, Sigrid A Langhans

**Affiliations:** 1 Nemours/Alfred I. duPont Hospital for Children, Wilmington, DE 19803, USA; 2 Nemours/Alfred I. duPont Hospital for Children; Rockland Center I, 1701 Rockland Road, Wilmington, DE 19803, USA

## Abstract

**Background:**

Curcumin (diferuloylmethane), the yellow pigment in the Asian spice turmeric, is a hydrophobic polyphenol from the rhizome of *Curcuma longa*. Because of its chemopreventive and chemotherapeutic potential with no discernable side effects, it has become one of the major natural agents being developed for cancer therapy. Accumulating evidence suggests that curcumin induces cell death through activation of apoptotic pathways and inhibition of cell growth and proliferation. The mitotic checkpoint, or spindle assembly checkpoint (SAC), is the major cell cycle control mechanism to delay the onset of anaphase during mitosis. One of the key regulators of the SAC is the anaphase promoting complex/cyclosome (APC/C) which ubiquitinates cyclin B and securin and targets them for proteolysis. Because APC/C not only ensures cell cycle arrest upon spindle disruption but also promotes cell death in response to prolonged mitotic arrest, it has become an attractive drug target in cancer therapy.

**Methods:**

Cell cycle profiles were determined in control and curcumin-treated medulloblastoma and various other cancer cell lines. Pull-down assays were used to confirm curcumin binding. APC/C activity was determined using an *in vitro *APC activity assay.

**Results:**

We identified Cdc27/APC3, a component of the APC/C, as a novel molecular target of curcumin and showed that curcumin binds to and crosslinks Cdc27 to affect APC/C function. We further provide evidence that curcumin preferably induces apoptosis in cells expressing phosphorylated Cdc27 usually found in highly proliferating cells.

**Conclusions:**

We report that curcumin directly targets the SAC to induce apoptosis preferably in cells with high levels of phosphorylated Cdc27. Our studies provide a possible molecular mechanism why curcumin induces apoptosis preferentially in cancer cells and suggest that phosphorylation of Cdc27 could be used as a biomarker to predict the therapeutic response of cancer cells to curcumin.

## Background

Curcumin, or diferuloylmethane, is a hydrophobic polyphenol derived from the rhizome of the herb *Curcuma longa*. It is better known as the yellow pigment in the widely used Asian spice turmeric. Recently, curcumin gained attention as an anti-cancer agent because of its chemopreventive and chemotherapeutic potential while having no discernable side effects. Curcumin induces apoptosis in various tumor cells and can prevent tumor initiation and growth in carcinogen-induced rodent models as well as in subcutaneous and orthotopic tumor xenografts [1-3]. Although it is still not known why curcumin preferentially kills tumor cells, it has been identified as one of the major natural agents that inhibit tumor initiation and tumor promotion.

Curcumin inhibits the proliferation of a wide variety of cancer cells including breast, blood, colon, liver, pancreas, kidney, prostate, and skin [1,2]. We and others have shown that it induces cell death in medulloblastoma, the most common pediatric brain tumor [3-5], and inhibits tumor growth in *in vivo *medulloblastoma models [3]. Curcumin has been suggested to selectively target tumor cells by affecting signaling pathways that regulate cell growth and survival and thus preferably induces apoptosis in highly proliferating cells [6,7]. Accumulating evidence suggests that curcumin-induced cell death is mediated both by the activation of cell death pathways and by the inhibition of growth/proliferation pathways [6,7]. Cell cycle regulatory proteins and checkpoints are downstream elements of cellular signaling cascades crucial for cell proliferation. Curcumin exerts various effects on cell cycle proteins and checkpoints, including p53, cyclin D1, cyclin dependent kinases (CDK), and CDK inhibitors (CDKi) such as p16^INK4a^, p21^WAF1/CIP1^, and p27^KIP1 ^[8]. It most often induces G2/M arrest, although G0/G1 arrest has been found in some cells [7]. It is well accepted that a prolonged arrest in G2/M phase leads to apoptotic cell death [9,10]. However, how curcumin induces G2/M arrest is not well understood.

The mitotic checkpoint, also known as the spindle assembly checkpoint (SAC) is the major cell cycle control mechanism in mitosis and delays the onset of anaphase until each single kinetochore has become attached to the mitotic spindle [10]. At the molecular level, the SAC is a signaling pathway consisting of multiple components that communicate between local spindle attachment and global cytoplasmic signaling to delay segregation. One of the key regulators of the SAC is the anaphase promoting complex/cyclosome (APC/C), an E3 ubiquitin ligase. In humans, the APC/C is a multi-protein complex consisting of at least 12 different subunits that requires other cofactors for proper functioning; a ubiquitin-activating (E1) enzyme, a ubiquitin-conjugating (E2) enzyme and co-activator proteins Cdc20 or Cdh1 [11,12]. Upon activation, APC/C ubiquitinates cyclin B and securin and targets them for destruction by proteolysis allowing for mitotic exit [11,12]. However, APC/C is not only a major effector of the SAC that ensures cell cycle arrest upon spindle disruption but it also promotes cell death upon prolonged mitotic arrest [10]. Thus, APC has become an attractive drug target to control the growth and proliferation of cancer cells and facilitate their apoptotic death.

Curcumin has a diverse range of molecular targets, including thioredoxin reductase, cyclooxygenase-2 (COX-2), protein kinase C, 5-lipoxygenase (5-LOX), and tubulin [6], supporting the concept that it may act upon numerous biochemical and molecular cascades. One interesting feature of curcumin is its ability to crosslink proteins such as the cystic fibrosis chloride channel (CFTR) thereby activating the channel [13]. In this study, we provide evidence that Cdc27, a component of the APC/C is a novel target for curcumin and that curcumin binds and crosslinks Cdc27. We also show that curcumin inhibits APC/C activity suggesting that curcumin binding to Cdc27 might play an important role in prolonged G2/M arrest induced apoptosis. In addition, curcumin preferentially induced apoptosis in cells progressing through G2/M and expressing phosphorylated Cdc27 usually found in highly proliferating cells. Thus, our studies reveal that the SAC is a molecular target of curcumin and, in addition, provide a possible explanation for why curcumin preferably induces cell death in cancer cells as previously reported [6,7].

## Methods

### Cell lines and reagents

All cell lines were obtained from the American Type Culture Collection (ATCC, Manassas, VA) and cultured according to ATCC protocols. The human medulloblastoma cell line DAOY was cultured in MEM supplemented with 10% fetal bovine serum, glutamine and penicillin/streptomycin in a humidified, 5% CO_2 _atmosphere at 37°C.

Antibodies against α-tubulin, acetylated α-tubulin, cleaved caspase3, cleaved PARP, GAPDH, cyclin A, and cyclin D1 and horseradish peroxidase (HRP)-conjugated secondary antibodies were obtained from Cell Signaling Technology (Danvers, MA), APC2, APC7, and APC8 from Biolegend (San Diego, CA) and Cdc27, Cdc20, BubRI, and β-actin from BD Transduction Laboratories (Franklin Lakes, NJ). Antibody against cyclin B1 was purchased from Santa Cruz Biotechnology (Santa Cruz, CA) and securin from Abcam (Cambridge, MA). Cdh1 and cyclin E antibodies, curcumin and half-curcumin (dehydrozingerone, DHZ, 4-(4-hydroxy-3-methoxyphenyl)-3-buten-2-one) were purchased from Sigma-Aldrich (St. Louis, MO).

### Cytotoxicity assay

Lactate dehydrogenase (LDH) levels as a measure of cell death were determined using the Non-radioactive Cytotoxicity kit (Promega, Madison, WI) according to manufacturer's instructions. LDH release was determined from curcumin-treated and untreated control cells grown on 24-well plates by collecting growth medium. Cell debris was removed by centrifugation. Viable cell LDH was collected from cells lysed by freezing for 15 min at -70°C followed by thawing at 37°C. The medium was collected and cleared from cell debris by centrifugation. The relative release of LDH was determined as the ratio of released LDH versus total LDH from viable cells.

### Immunoblotting, immunoprecipitations, and λ-phosphatase treatment

Cell lysates were prepared in a buffer containing 20 mM Tris (pH 7.5), 150 mM NaCl, 1 mM EDTA, 1 mM EGTA, 0.1% Triton X-100, 2.5 mM sodium pyrophosphate, 1 mM β-glycerolphosphate, 1 mM sodium vanadate, 1 mM phenylmethylsulfonyl fluoride and 5 μg/ml of antipapain, leupeptin and pepstatin (protease inhibitor cocktail). Protein concentrations were determined by the Dc protein assay (Bio-Rad, Hercules, CA). Equal amounts of protein were resolved by SDS-PAGE and transferred to nitrocellulose. The membranes were blocked in 5% non-fat milk in Tris-buffered saline with 0.1% Tween-20 (TBST). Primary antibodies diluted in 5% bovine serum albumin/TBST were incubated overnight at 4°C and HRP-conjugated secondary antibodies in 5% non-fat milk/TBST for 2 h at room temperature. Protein bands were visualized by Enhanced Chemiluminescene Plus (GE Healthcare, Piscataway, NJ).

For immunoprecipitation, cells were lysed at 4°C for 30 min in a buffer of 50 mM HEPES, pH 7.4, 150 mM NaCl, 0.5% NP-40, 1 mM EDTA, 1 mM Na_3_VO_4_, 1 mM aprotinin, 1 mM leupeptin and 1 mM PMSF. Equal amounts of protein (from 0.5 to 2 mg) were incubated with Cdc27 antibody for 4 h at 4°C followed by protein G-sepharose (GE Healthcare) for 2 h, washed extensively, and analyzed by immunoblotting with indicated antibodies. For λ-phosphatase treatment, Cdc27 was immunoprecipitated as above except that phosphatase and protease inhibitors were omitted and then incubated with λ - phosphatase according to the manufacturer's protocol (New England Biolabs, Ipswich, MA).

### Cell cycle analysis

Interphase DAOY cells were treated with curcumin for indicated times, trypsinized, and fixed in cold 70% ethanol. DNA was stained with 100 μg/ml propidium iodide (PI) in hypotonic citrate buffer with 20 μg/ml ribonuclease A. Stained nuclei were analyzed for DNA-PI fluorescence using an Accuri C6 flow cytometer (Accuri Cytometers Inc., Ann Arbor, MI). Resulting DNA distributions for sub G0/G1, G0/G1, S and G2/M phase of the cell cycle were analyzed with CFlow plus software (Accuri Cytometers Inc).

For analysis of cell cycle profiles after mitotic block, cells were synchronized with 2 mM thymidine for 24 h. The block was released for 3 h and cells were arrested in prometaphase with 100 nM nocodazole for 12 h, resulting in approximately 70% of the cells arrested in G2/M. For G1/S arrest, cells were synchronized for 18 h with 2 mM thymidine, released for 9 h, followed by a second thymidine arrest for 18 h, resulting in a G1/S block in about 50% of the cells. The block was then released in the presence of DMSO or curcumin as indicated and the cells were processed as described above.

### *In vitro *APC assay

*In vitro *APC assays were performed as described [14] using an *in vitro *transcribed and translated N-terminal fragment of cyclin B_1 _(cyclin B_1_-N_1-102_) as substrate. ^35^S-methionine labeled cyclin B_1_-N_1-102 _was obtained using the TNT quick-coupled Transcription/Translation system (Promega, Madison, WI). Cell pellets of control and curcumin-treated DAOY cells were snap frozen in liquid nitrogen. The cell pellets were resuspended in an ice-cold hypotonic buffer (20 mM Hepes pH 7.6, 20 mM NaF, 1.5 mM MgCl_2_, 1 mM DTT, 5 mM KCl, 20 mM β-glycerophosphate, 250 μM NaVO_3_, 1 mM PMSF, and EDTA-free protease inhibitors) and incubated for 30 min on ice. The lysates were briefly homogenized and cleared by a 1 h centrifugation at 13,000 rpm in a micro centrifuge. For the assay, 30 μg of total protein were added to reaction buffer containing 20 mM Tris pH 7.5, 20 mM NaCl, 5 mM MgCl_2_, 5 mM ATP-γ-S, 20 μg/ml MG-132, 0.5 μg UbcH10, 20 μM ubiquitin, 1 μm ubiquitin aldehyde, protease inhibitors, and 2 μl of *in vitro *translated^35^S-cyclin B_1_-N_1-102 _and incubated at 37°C for 60 min. The reactions were stopped by adding sample buffer and proteins were separated by SDS-PAGE on a 4-15% gradient gel. To visualize the bands, the gel was incubated and enhanced with salicylate, dried, and then subjected to autoradiography.

### Immobilization of curcumin on epoxy-activated Sepharose 6B

Curcumin was coupled to epoxy-activated Sepharose 6B as previously described [15]. Briefly, 20 mM curcumin dissolved in coupling buffer (50% dimethylformamide/0.1 M Na_2_CO_3_/10 mM NaOH) was incubated with swollen epoxy-activated Sepharose 6B beads overnight at 30°C. After washing, unoccupied binding sites were blocked with 1 M ethanolamine by overnight incubation. Low (0.1 M acetate buffer, pH 4) and high (0.1 M Tris-HCl, pH 8, 0.5 M NaCl) pH buffers were used each three times to wash and equilibrate the beads. Control beads were prepared in parallel with curcumin-coupled beads but curcumin was omitted. DAOY cell lysates were prepared in a lysis buffer of 100 mM HEPES, pH 7.6, 300 mM NaCl, 0.1% Triton X-100, 2 mM EDTA, 2 mM EGTA supplemented with phosphatase and protease inhibitors. 500 μg of protein was mixed with 20 μl of curcumin-coupled Sepharose beads and incubated for 3 h at 4°C. After washing bound proteins were eluted with 1× SDS-PAGE sample buffer and processed for immunoblotting.

### Statistical analysis

Data are presented as mean ± SD unless otherwise indicated. The differences between means of two groups were analyzed by a two-tailed unpaired Student's *t*-test. When required, P values are stated in the figure legends.

## Results

### Curcumin induced cell death is cell cycle dependent

**Figure 1:**
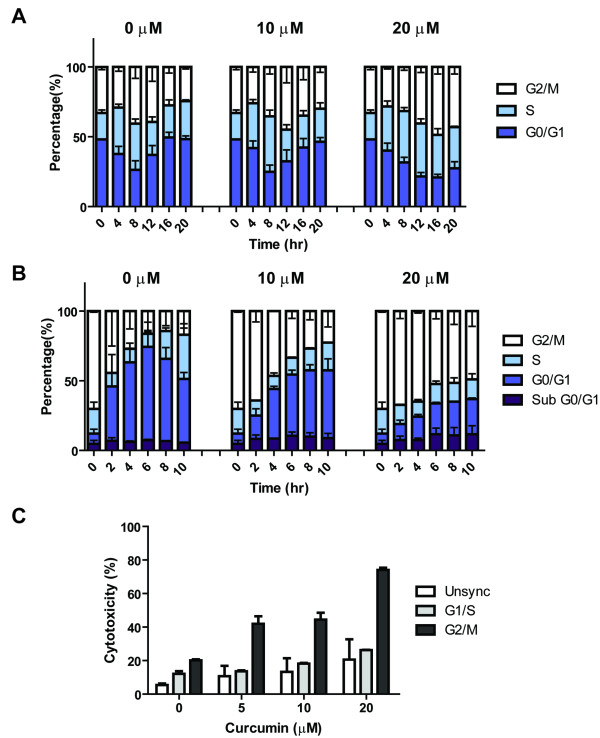
**Curcumin induced cell cycle arrest in medulloblastoma cells**. **A**, DAOY cells were arrested in G1/S and the block was released in the presence of 0, 10 or 20 μM curcumin. At indicated time points cell cycle progression was analyzed by flow cytometry. **B**, Mitotically arrested DAOY cells were released from their block in the presence of 0, 10 or 20 μM curcumin. Cell cycle profiles were determined at the indicated time points. **C**, Unsynchronized cells were treated with indicated concentrations of curcumin for 12 h. G1/S-arrested DAOY cells were released from their block for 12 h in the presence of curcumin. To obtain G2/M arrested cells, DAOY cells were synchronized first at G1/S. The block was released for 8 h in the absence of any drugs. Cells were then treated with the indicated concentration of curcumin for an additional 12 h. The cell-cycle dependent cytotoxicity of curcumin was measured by an LDH assay. **A-C**, The data are the mean ± SEM of three independent experiments.

Curcumin can arrest cell-cycle progression and induce apoptosis in various cancer cells [7]. We and others reported previously that curcumin induces G2/M arrest and apoptosis in medulloblastoma cells [3-5]. We further found that DAOY medulloblastoma cells released from a G1/S block in the presence of curcumin progressed much slower through the cell cycle compared to vehicle-treated control cells (Figure 1A and Additional file 1: Table S1). While most control cells reached G2/M 8-12 h after release and almost all of G1/S blocked cells re-entered G0/G1 after 16 h, cells released in the presence of 10 and 20 μM curcumin reached G2/M only after 12-16 and 16-20 h, respectively. In addition, 56.9% of the cells released in the presence of 20 μM curcumin had not re-entered G0/G1 even 20 h after removal of the thymidine block. However, no sub-G0/G1 signal was detected indicating that although the cells were delayed in mitosis they did not undergo apoptosis within this time frame (Figure 1A). We also arrested DAOY cells in G2/M by thymidine/nocodazole treatment and released the block in the presence or absence of curcumin (Figure 1Band Additional file 2: Table S2). While 70.2% of the cells were blocked in G2/M, 36.8% of control cells exited mitosis within 2 hours of release and by 6 h 76.9% had exited G2/M. In the presence of 10 μM curcumin mitotic exit was significantly delayed and after 2 and 6 hours 91.5% and 47.7% of the cells, respectively, remained in G2/M. This effect was much more pronounced in the presence of 20 μM curcumin when after 10 h of release still 69.8% of the cells were found in G2/M. At the same time a significant amount of cells was in the sub-G0/G1 fraction suggesting that curcumin-induced delay from G2/M exit may commit the cells to undergo apoptosis. Together these data suggest that the sensitivity of DAOY cells to curcumin-induced cell-death might be cell-cycle dependent. Indeed, DAOY cells treated with 20 μM curcumin in G2/M were 3-fold more sensitive to curcumin-induced cell death than cells arrested in either G1/S or unsynchronized control cells (Figure 1C). Thus, curcumin may affect the function of proteins directly involved in G2/M progression to ultimately induce cell death.

### Curcumin binds to the Cdc27/APC3 subunit of APC/C

**Figure 2:**
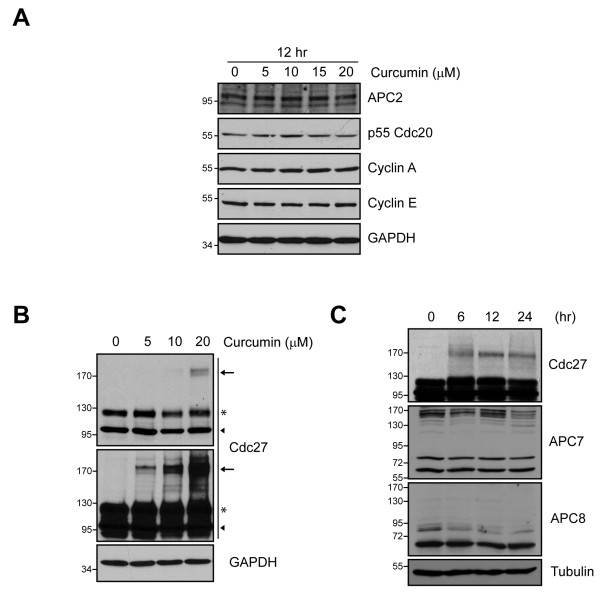
**Curcumin-induced effects on APC/C and other cell cycle related proteins**. **A**, Expression of the APC/C subunit APC2, the APC/C co-activator p55Cdc20 and cyclins A and E in control and curcumin-treated DAOY cells as determined by immunoblotting. GAPDH levels are included to ensure equal loading. **B**, Immunoblot of the APC/C subunit Cdc27 showing a curcumin-induced shift in MW (arrows) in unsynchronized DAOY cells. Because of differences in band intensity the same immunoblot is shown with two different exposures. Arrowhead and asterisk indicate the non-phosphorylated and phosphorylated bands of Cdc27, respectively **C**, Comparison of MW shift by curcumin between Cdc27 and other APC components. Tubulin was used for equal loading control.

**Figure 3:**
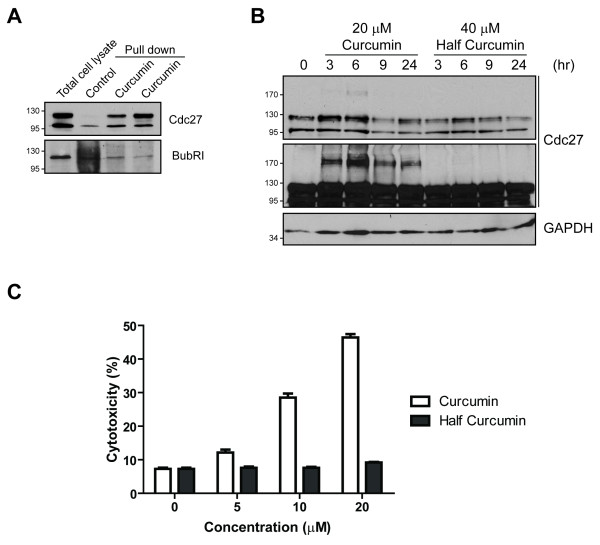
**Curcumin associates with Cdc27**. **A**, DAOY cell lysates were incubated with curcumin-bound sepharose beads and then subjected to SDS-PAGE and immunoblotting with Cdc27. A BubR1 immunoblot was included as control for non-specific binding. **B**, DAOY cells were incubated with either curcumin or half-curcumin for indicated time points. Cell lysates were separated by SDS-PAGE and immunoblotted for Cdc27. The middle panel shows a longer exposure of the same blot in the upper panel. **C**, LDH release as a measure of cytotoxicity of curcumin and half-curcumin in DAOY cells treated for 16 h. Data represent mean ± SEM of three independent experiments.

To test whether curcumin affects known regulators of mitosis, we analyzed the expression of various cell cycle proteins in control and curcumin-treated DAOY cells. We found no noticeable changes in cyclin A and E that are major players in S-phase and G1/S transition, respectively. Also, the levels of APC2, an APC/C subunit essential for ubiquitination, or the APC/C co-activator p55Cdc20 were comparable in control and curcumin-treated cells (Figure 2A). Interestingly, immunoblots of Cdc27 revealed a high molecular weight (MW) band in curcumin-treated cells that was approximately double the MW of Cdc27 and its intensity increased with increasing curcumin concentrations (Figure 2B). This effect seemed to be specific for Cdc27 since a MW shift of APC7 or APC8 that both, like Cdc27/APC3, have TPR domains (Figure 2C), was not detectable. It has been shown that curcumin can affect a protein's function by direct cross-linking [13]. Thus, we tested whether curcumin could bind directly to Cdc27. Indeed, curcumin-bound sepharose beads from two independent preparations pulled down Cdc27 while it was barely detected with control beads (Figure 3A). In addition, half-curcumin which has only one β-diketone moiety and does not have cross-linking capacity [13], failed to induce the high MW bands of Cdc27, further suggesting that curcumin indeed induces the formation of Cdc27 dimers (Figure 3B). Interestingly, half-curcumin also failed to induce cell death in DAOY cells (Figure 3C) indicating that cross-linking of Cdc27 may be an essential step in curcumin-induced apoptosis in these cells (Figure 3C).

In addition, we consistently observed decreased levels of non-crosslinked Cdc27 in curcumin-treated cells (Figure 3B and data not shown). We recently showed that curcumin increases survival in Smo/Smo mice, a transgenic medulloblastoma mouse model, and reduces tumor growth of DAOY xenografts [3]. Interestingly, we found that in tumors from curcumin-treated mice, the Cdc27 levels were reduced (Additional file 3: Figure S1 and our unpublished data) when compared with control mice. However, we were not able to detect the high MW Cdc27 characteristic for crosslinking, which could be due to the lower Cdc27 levels found in these tumors per se. Nevertheless, it suggests the possibility that curcumin targets Cdc27 *in vivo *to reduce tumor growth.

### Cdc27 phosphorylation sensitizes tumor cells to curcumin

**Figure 4:**
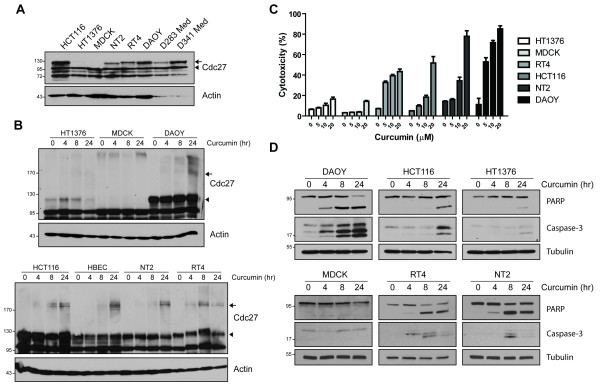
**Cdc27 phosphorylation sensitizes tumor cells to curcumin**. **A**, Immunoblot of Cdc27 in various cell lines, DAOY, NT2, D283 Med, D341 Med (brain); HCT116 (colon); HT1376, RT4 (bladder); MDCK (kidney). The arrow indicates the band corresponding to phosphorylated Cdc27 and arrowhead shows unphosphorylated Cdc27. Actin immunoblot is shown as loading control. **B**, Cdc27 immunoblot of cell lines in (**A**) after treatment with 20 μM curcumin for indicated times. Arrows indicate crosslinked Cdc27, while arrowheads show phosphorylated Cdc27. Equal amounts of protein were used as shown by actin immunoblot. **C**, Cytotoxicity of curcumin in six different cell lines with different Cdc27 phosphorylation levels. LDH release was determined after 24 h of exposure to curcumin at indicated concentrations. Data are the mean ± SEM of three independent experiments. **D**, Immunoblots of cleaved PARP and caspase-3 as indicators of apoptosis upon exposure to 20 μM curcumin. Tubulin immunoblot ensures equal amounts of protein being used for analysis. Arrows indicate cleaved PARP.

In pull-down assays we observed that curcumin seemed to have a higher affinity for the 130 kDa form of Cdc27 (Figure 3A, upper band). As reported earlier, this MW is consistent with the phosphorylated form of Cdc27 which we confirmed by λ-phosphatase treatment (Additional file 4: Figure S2). To test whether phosphorylation of Cdc27 is associated with increased sensitivity to curcumin-induced cell death, we first screened several cell lines for Cdc27 phosphorylation (Figure 4A). Interestingly, only in cell lines with the phosphorylated form of Cdc27 was curcumin able to crosslink Cdc27 (Figure 4B) further confirming that curcumin dimerizes preferentially phosphorylated Cdc27. We then chose six of these cell lines with high (DAOY, HCT116), intermediate (RT4, NT2) and low (HT1376, MDCK) levels of phosphorylated Cdc27 and tested their sensitivity to curcumin-induced cell death. As expected DAOY cells were most sensitive to curcumin-induced apoptosis while MDCK and HT1376 cells were almost unaffected (Figure 4C, D; Additional file 5: Figure S3), suggesting that curcumin preferentially induces apoptosis in cells with high levels of Cdc27 phosphorylation.

### Curcumin inhibits APC activity

**Figure 5:**
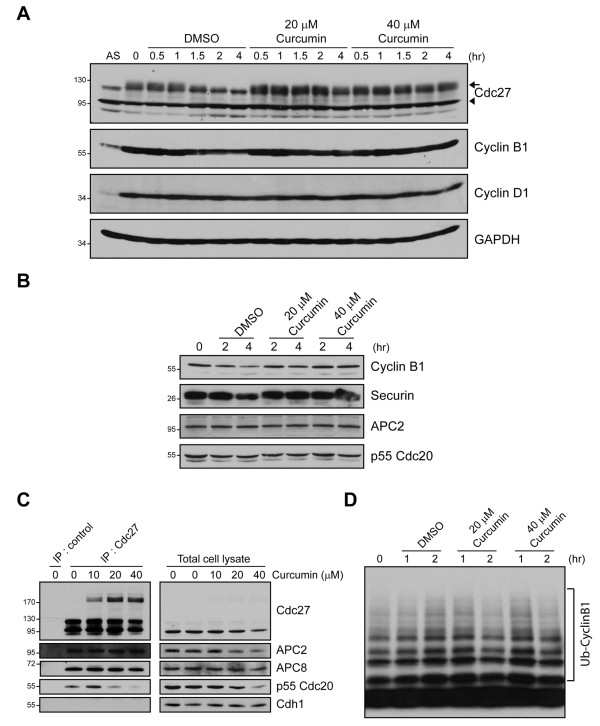
**Curcumin inhibits APC/C activity**. **A**, DAOY cells were released from thymidine/nocodazole block in the presence of 0, 20 and 40 μM curcumin for indicated time points. Cell lysates were subjected to immunoblotting with antibodies shown. Arrow indicates phosphorylated Cdc27, while the arrowhead shows unphosphorylated Cdc27. **B**, Mitotically arrested DAOY cells were released with different concentrations of curcumin for 2 and 4 h, respectively, and blotted with antibodies indicated. **C**, DAOY cells were synchronized by double thymidine arrest and then incubated with curcumin for 8 h. Cell lysates were subjected to immunoprecipitation with anti-Cdc27 antibodies. Immunoprecipitated proteins were immunoblotted with antibodies indicated. Immunoblots of total cell lysates are shown to ensure equal loading **D**, Mitotically arrested DAOY cells were released in the presence of either DMSO or curcumin for indicated time points and APC/C activity was determined as described in Materials and Methods.

Many APC/C components are phosphorylated during mitosis, which seems to be required for APC/C activity [16,17]. To test whether cross-linking of Cdc27 by curcumin compromises APC/C activity, we arrested DAOY cells in G2/M and released the block in the absence or presence of curcumin. Release of the mitotic block in DMSO-treated control cells resulted in the dephosphorylation of Cdc27 over time which was not observed in curcumin-treated cells (Figure 5A). In addition, decreases in the cyclin B1 and securin levels that are a prerequisite for mitotic exit were not found in curcumin-treated cells but were readily observed in control cells (Figures 5A, B). In contrast, no significant differences were found in the levels of the core APC/C subunit APC2, the APC/C coactivator p55Cdc20 or cyclin D1 in control and curcumin-treated cells (Figures 5A, B). Together, these data suggest that curcumin might directly affect the function of the APC/C.

Proper APC/C function requires co-activator proteins such as Cdc20 or Cdh1 that may facilitate the recruitment of substrates. Co-immunoprecipitation analysis in DAOY cells released from a G2/M block in the presence of curcumin showed that p55Cdc20 association with Cdc27 was dramatically reduced compared to control cells while the Cdc27 association with the APC/C subunits APC2 and APC8 was not affected (Figure 5C). Under the experimental conditions used we did not find Cdh1 associating with Cdc27 (Figure 5C). We next tested whether curcumin affects the activity of APC/C using an *in vitro *APC assay that monitors APC's ubiquitin ligase activity on cyclin B as described earlier [14]. The cells were arrested in G2/M and released from the block in the presence or absence of curcumin. Compared to cells blocked at G2/M (Figure 5D), we found a gradual increase of APC activity upon block release in control cells indicating that these cells were exiting mitosis. In contrast, in curcumin-treated cells the APC activity was reduced 2 hours after block release when compared to cells after one hour of release indicating that curcumin inhibits APC activity directly. Together these data suggest that cross-linking of Cdc27 by curcumin reduces its association with its co-activator p55Cdc20 thus inhibiting APC activity.

## Discussion

In recent years many targets of curcumin have been identified, but the molecular mechanism how curcumin induces cell cycle arrest at G2/M remains elusive. In this study, we provide evidence that curcumin could directly target the SAC to inhibit progression through mitosis. We show that curcumin binds to and crosslinks Cdc27, a component of the APC/C and critical for its function. Consistent with this, we found that curcumin inhibits APC/C activity thereby preventing the degradation of cyclin B1 and securin, consequently inducing G2/M arrest. Furthermore, curcumin appeared to have a greater affinity for phosphorylated Cdc27, which is usually found in mitotically active cells. Cell lines that had little or no phosphorylated Cdc27 thus were less sensitive to curcumin-induced apoptosis. These results could provide an explanation why cancer cells are more sensitive than normal cells to curcumin-induced cell death and suggest that phosphorylated Cdc27 might have the potential to be developed as biomarker for effective curcumin-based therapy in cancer.

### Curcumin crosslinks the APC subunit cdc27

Curcumin affects a multitude of molecular targets including transcription factors, receptors, kinases, inflammatory cytokines, and other enzymes (for a comprehensive review see [18]). It modulates multiple signaling pathways including pathways involved in cell proliferation (cyclin D1, c-myc), cell survival (Bcl-2, Bcl-xL, cFLIP, XIAP, c-IAP1), and apoptosis (caspase-8, 3, 9). Other pathways affected by curcumin include those comprising protein kinases (JNK, Akt, AMPK), tumor suppressors (p53, p21), death receptors (DR4, DR5), mitochondrial pathways and endoplasmic reticulum stress responses. Curcumin has also been shown to alter the expression and function of COX2 and 5-LOX at the transcriptional and post-translational levels. Thus, it is possible that many of the cellular and molecular effects observed in curcumin treated cells might be due to downstream effects rather than direct interactions with curcumin.

Although there are now a multitude of studies on curcumin's cellular effects, surprisingly little is known about the direct interactions of curcumin with its target molecules. One of the better characterized interactions is the binding of curcumin to CFTR [13]. Curcumin can crosslink CFTR polypeptides into SDS-resistant oligomers in microsomes and in intact cells. However, the ability of curcumin to rapidly and persistently stimulate CFTR channels was unrelated to the crosslinking activity. Interestingly, we found that curcumin can bind to Cdc27 *in vitro *and can crosslink Cdc27 in a variety of cell lines. While CFTR channel activation was unrelated to the cross-linking of CFTR, we found evidence that crosslinking of Cdc27 by curcumin appeared to affect Cdc27 functions itself; half-curcumin neither crosslinked Cdc27 nor induced apoptosis in DAOY cells.

However, at this point it is not known how curcumin crosslinks Cdc27 and affects its function. Bernard [13] suggested that curcumin possibly reacts with the CFTR through an oxidation reaction involving the reactive β-diketone moiety. Since half-curcumin that has only one β-diketone moiety did not crosslink CFTR, the authors further concluded that the symmetrical structure of curcumin is required for crosslinking and that crosslinking might occur within one CFTR molecule. Similarly, we found that half-curcumin failed to crosslink Cdc27 indicating that Cdc27 crosslinking also requires the symmetrical structure of curcumin. Interestingly, increasing evidence suggests that Cdc27 exists as a homo-dimer within APC/C and that this dimerization is essential for its function. It is possible that curcumin chemically crosslinks dimerized Cdc27 within the APC complex, thus interfering with its function.

While curcumin was able to bind to both unphosphorylated and phosphorylated Cdc27, we observed that only cells expressing phosphorylated Cdc27 showed the shift to the high molecular weight Cdc27. In addition these cells were more susceptible to curcumin induced cell death. It is possible that phosphorylation induces conformational changes that are more permissive for curcumin binding and/or crosslinking of the protein and thus curcumin is more effective in these cells. Cdc27 is one of the five APC subunits with tetratrico-peptide repeats (TPR). Nevertheless, we did not find any crosslinking of other APC subunits with the TPR motif, suggesting that curcumin crosslinking is specific to Cdc27. Thus, identification of curcumin's binding motifs will not only be important to understand curcumin's biological roles but also will be a major step in developing more specific and effective curcumin analogs for therapy.

### Curcumin impedes the interaction of Cdc27 and the APC/C activator p55Cdc20

Cdc27 is considered as a core component of the APC/C that secures the interaction with substrate/coactivator complexes [19]. It directly binds activator subunits such as p55Cdc20 or cdh1 and associates with mitotic checkpoint proteins including Mad2 and BubR1 [20]. Consistent with a role of Cdc27 in controlling the timing of mitosis and the notion that curcumin-mediated crosslinking of Cdc27 impairs its function, we observed a delay in the mitotic exit in curcumin-treated cells when compared to control cells. It is thought that the SAC acts by inhibiting the p55Cdc20-bound form of the APC/C and that repression of APC/C stabilizes its downstream targets including cyclin B and securin [11,21]. We not only found that curcumin treatment blocked cyclin B1 and securin degradation but also observed a decreased association of p55Cdc20 with Cdc27 under these conditions. At the same time, association of Cdc27 with other subunits of the APC/C such as APC2 and APC8 did not change (Figure 5C). Thus, we suggest that curcumin might repress APC/C function by preventing the efficient association of the APC/C core complex with its activator p55Cdc20.

APC/C is partially activated through phosphorylation of core subunits. Cdc27 undergoes mitosis-specific phosphorylation [22,23] which seems to enhance the affinity between APC/C and p55Cdc20 thereby ensuring its activation [24-27]. Analysis of mitosis-specific phosphorylation sites in Cdc27 revealed that most of them are clustered in confined regions, mainly outside of the TPR repeats [25]. We found that curcumin specifically crosslinks Cdc27 and not other APC/C subunits with TPR motifs. We also noticed that curcumin preferably binds to phosphorylated Cdc27 and induces apoptosis more effectively in mitotic cells. At this point we do not know how curcumin prevents p55Cdc20 binding to Cdc27. It is possible that curcumin blocks the phosphorylated interaction sites directly or that curcumin crosslinking induces a conformational change in Cdc27 that is less permissive to p55Cdc20 binding. It is also conceivable that curcumin binding to Cdc27 itself presents a steric hindrance for p55Cdc20 to access its binding sites. Whatever the mechanism, curcumin's interaction with mitotic phosphorylated Cdc27 might provide a possible explanation why curcumin preferentially induces cell death in tumor cells that are usually highly proliferative and not in normal cells [6,7].

### Curcumin-treatment induces tubulin acetylation

Curcumin has been reported to bind to tubulin, inhibit tubulin polymerization *in vitro*, depolymerize interphase and mitotic microtubules in HeLa and MCF-7 cells, and suppress the dynamic instability of microtubules in MCF-7 cells [28,29]. Microtubules form the mitotic spindle during cell division and because of the rapid assembly and disassembly of microtubules during the alignment and separation of chromosomes, spindle microtubules are highly dynamic [30]. We recently reported that mitotic spindle tubules in curcumin treated DAOY cells were disorganized and showed increased staining, suggestive of microtubule stabilization [3]. We also found that curcumin treatment increased tubulin acetylation in these cells. While the exact function of tubulin acetylation has not yet been determined, it is usually associated with increased microtubule stability [31]. Because of the discrepancies of the role of curcumin in tubulin depolymerization in interphase cells and tubulin stabilization in mitotic cells we had previously suggested that factors other than direct binding of curcumin to tubulin might contribute to the altered mitotic spindle organization in curcumin-treated cells [3]. Interestingly, it has been reported recently that p55Cdc20 interacts with histone deacetylase (HDAC) 6 [32]. HDAC6 can associate with microtubules and deacetylate α-tubulin [33]. At this point, we do not know whether there is a connection between reduced binding of p55Cdc20 to curcumin-crosslinked Cdc27, HDAC6 function, and tubulin acetylation. However, we found that in cells with low levels of phosphorylated Cdc27 in which curcumin failed to cross-link Cdc27 and that were less sensitive to curcumin treatment, curcumin-induced tubulin acetylation was also reduced (Additional file 6: Figure S4). Thus, loss of Cdc27 function or p55Cdc20 association with Cdc27 might be linked to increased tubulin acetylation in curcumin-treated cells.

### Cell cycle exit as a target for cancer therapy

The mitotic spindle is a validated target for cancer therapeutics. While antimitotic agents that target the mitotic spindle (such as vinca alkaloids, taxanes, and epothilones) are widely used in the clinic for the treatment of human malignancies they exhibit serious side-effects due to their effects on microtubule function in normal cells. In addition, upon activation of the SAC by a non-functional mitotic spindle, cells do not arrest in G2/M indefinitely. After an extended time of mitotic arrest, cells either die in mitosis by apoptosis or leak through the SAC by adaptation or mitotic slippage [9] which has been associated with resistance to antimitotic drugs [34]. Thus, blocking mitotic exit downstream of the checkpoint may be a better cancer therapeutic strategy than perturbing spindle assembly [35]. Indeed, Huang et al. [35] showed that blocking mitotic exit by p55Cdc20 knockdown induced cell death and suggested that a small molecule that binds APC/C and competes with the p55Cdc20 binding site might be the most obvious inhibition strategy. We suggest that curcumin might be such a small molecule that abrogates APC/C and p55Cdc20 interaction.

## Conclusions

We found that curcumin directly targets the SAC by binding to Cdc27, one of the core components of APC/C. Furthermore, we show that curcumin preferentially induces cell death in cells with phosphorylated Cdc27 and suggest that Cdc27 phosphorylation could be developed as a biomarker to identify curcumin-sensitive tumors. Although the *in vivo *bioavailability of curcumin is limited, many nanotechnology approaches are being developed for efficient curcumin delivery [1,36-38] and curcumin might prove to be an efficient drug to treat medulloblastoma and other cancers with minimal side effects.

## Abbreviations

APC/C: Anaphase promoting complex/cyclosome; CFTR: Cystic fibrosis chloride channel; CDK: Cyclin dependent kinases; CDKi: CDK inhibitor; LDH: Lactate dehydrogenase; SAC: Spindle assembly checkpoint.

## Competing interests

The authors declare that they have no competing interests.

## Authors' contributions

SJL designed and performed the research, analyzed the data and drafted the manuscript. SAL conceived the study, supervised the research, analyzed the data and drafted the manuscript. Both authors read and approved the final manuscript.

## Pre-publication history

The pre-publication history for this paper can be accessed here:

http://www.biomedcentral.com/1471-2407/12/44/prepub

## Supplementary Material

Additional file 1 **Table S1**. Curcumin blocks mitotic progression of DAOY cells arrested in G1/S. Quantitative analysis of data in Figure 1A. Click here for file

Additional file 2 **Table S2**. Curcumin blocks mitotic progression of DAOY cells arrested in G2/M. Quantitative analysis of data in Figure 1B. Click here for file

Additional file 3 **Figure S1**. Cdc27 levels in Smo/Smo mouse tumors. Immunoblot of Cdc27 levels in medulloblastoma samples obtained from Smo/Smo mice treated with curcumin or corn oil as previously described. Tubulin was shown for equal loading. Click here for file

Additional file 4 **Figure S2**. DAOY cells express hyperphosphorylated Cdc27. Immunoprecipitated Cdc27 was incubated with or without λphosphatase for indicated time points and then resolved in SDS-PAGE for Western blotting. Arrowhead indicates phosphorylated Cdc27 and asterisk indicates IgG of input antibodies. Click here for file

Additional file 5 **Figure S3**. Curcumin selectively blocks mitotic progression in different cell lines. NT2 and MDCK cells were arrested with thymidine/nocodazole treatment. Cells were washed and then released from mitotic block with different concentrations of curcumin for indicated time points. DNA contents were analyzed for cell cycle progression. Data are expressed as mean ± SEM of three independent experiments. Click here for file

Additional file 6 **Figure S4**. Curcumin-induced acetylated tubulin accumulation. Accumulation of acetylated tubulin in six different cell lines that were incubated with 20 μM curcumin for 0, 4, 8 and 24 h. Click here for file
