# Supplementary figures and images for: Anaphase-promoting complex/cyclosome protein Cdc27 is a target for curcumin-induced cell cycle arrest and apoptosis
Source: BMC Cancer. 2012 Jan 26;12:44. doi: 10.1186/1471-2407-12-44 (PMC3296673; doi:10.1186/1471-2407-12-44)

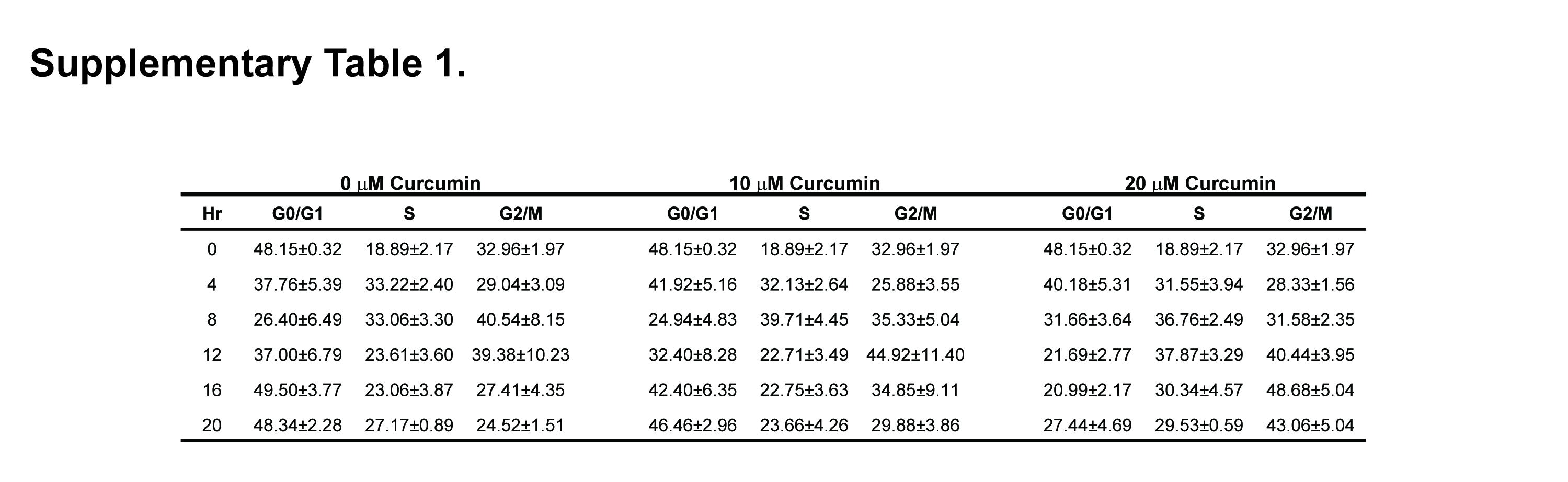

Supplement: Additional file 1 — Table S1. Curcumin blocks mitotic progression of DAOY cells arrested in G1/S. Quantitative analysis of data in Figure 1A. [file 1471-2407-12-44-S1.TIFF]

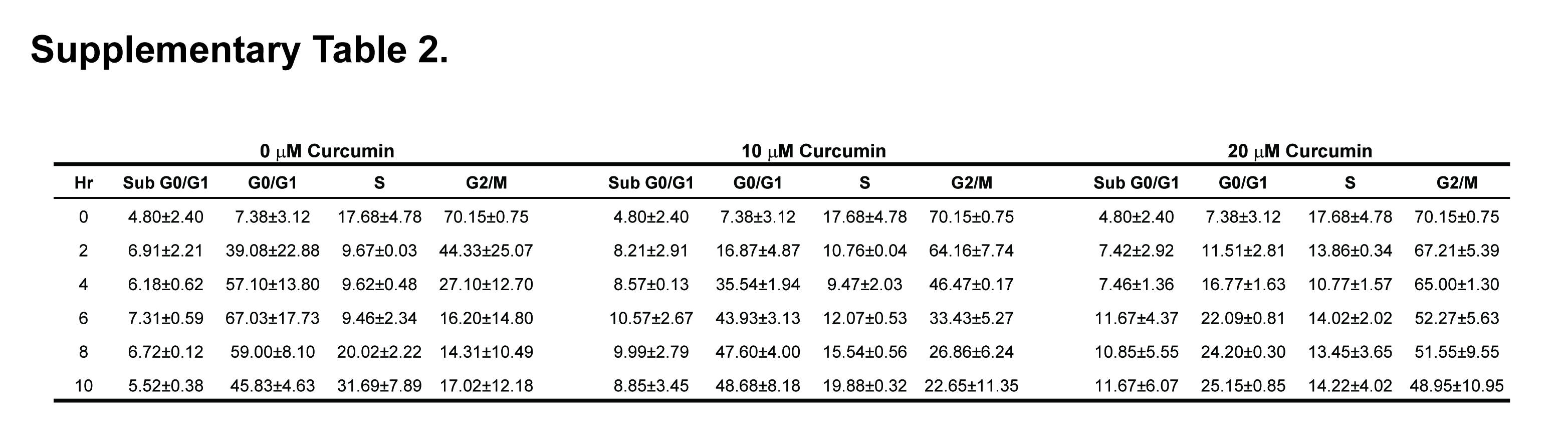

Supplement: Additional file 2 — Table S2. Curcumin blocks mitotic progression of DAOY cells arrested in G2/M. Quantitative analysis of data in Figure 1B. [file 1471-2407-12-44-S2.TIFF]

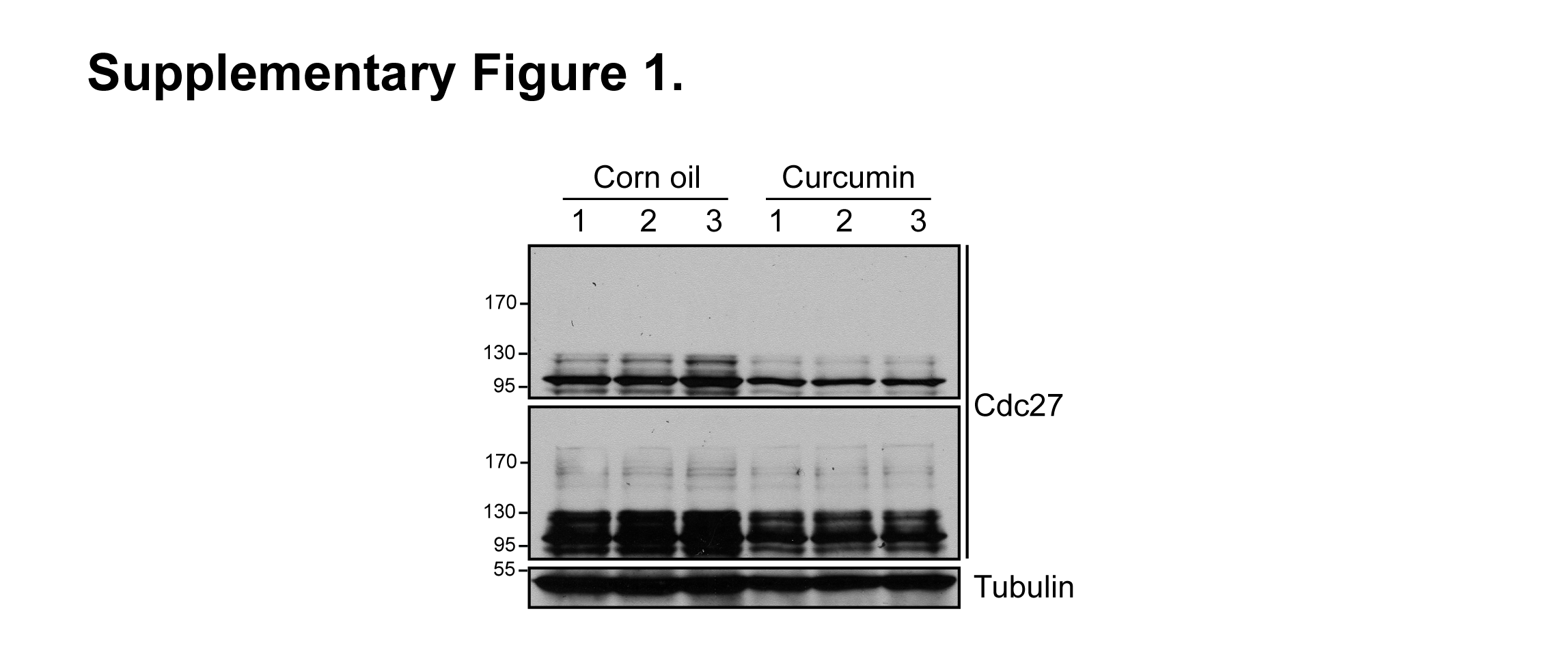

Supplement: Additional file 3 — Figure S1. Cdc27 levels in Smo/Smo mouse tumors. Immunoblot of Cdc27 levels in medulloblastoma samples obtained from Smo/Smo mice treated with curcumin or corn oil as previously described. Tubulin was shown for equal loading. [file 1471-2407-12-44-S3.TIFF]

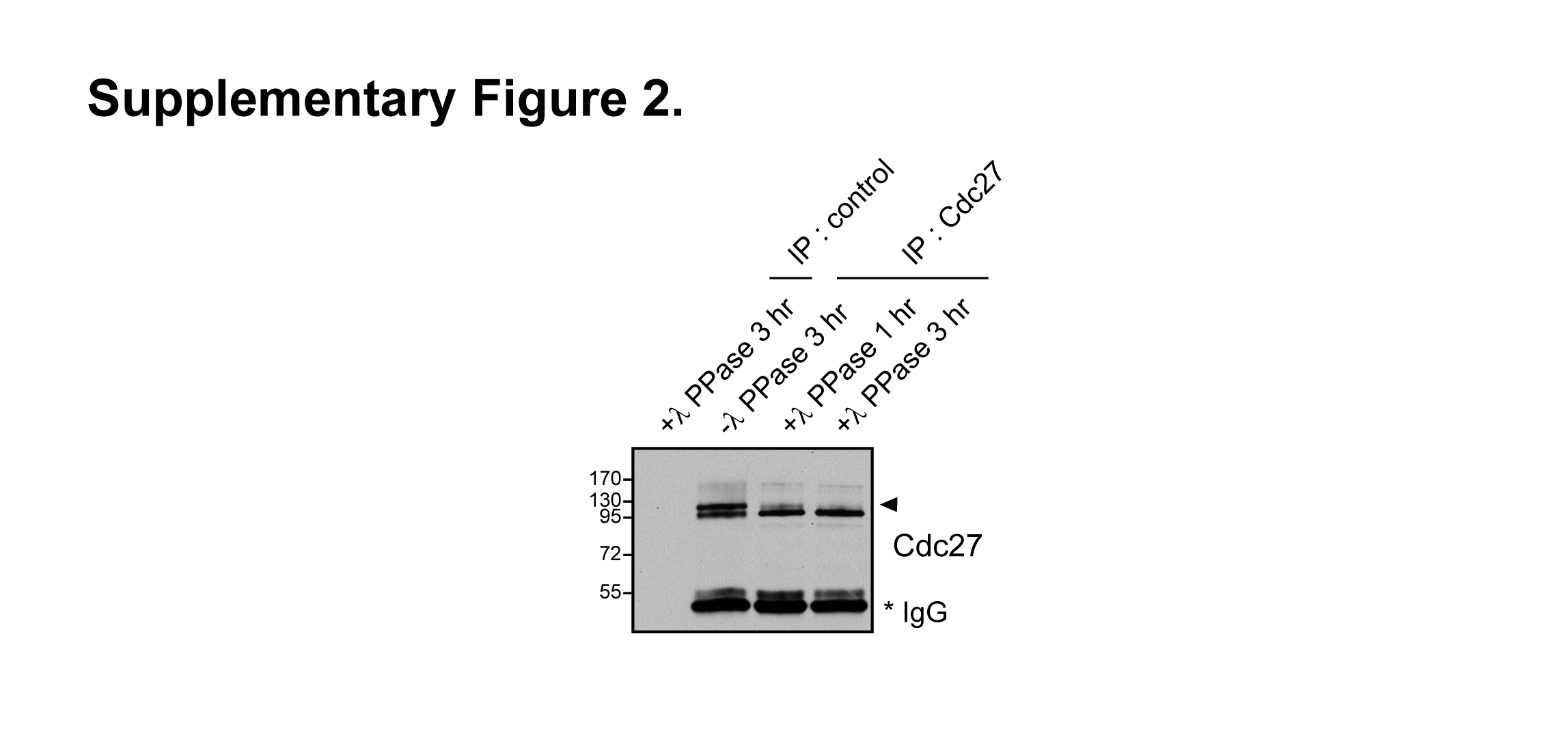

Supplement: Additional file 4 — Figure S2. DAOY cells express hyperphosphorylated Cdc27. Immunoprecipitated Cdc27 was incubated with or without λphosphatase for indicated time points and then resolved in SDS-PAGE for Western blotting. Arrowhead indicates phosphorylated Cdc27 and asterisk indicates IgG of input antibodies. [file 1471-2407-12-44-S4.TIFF]

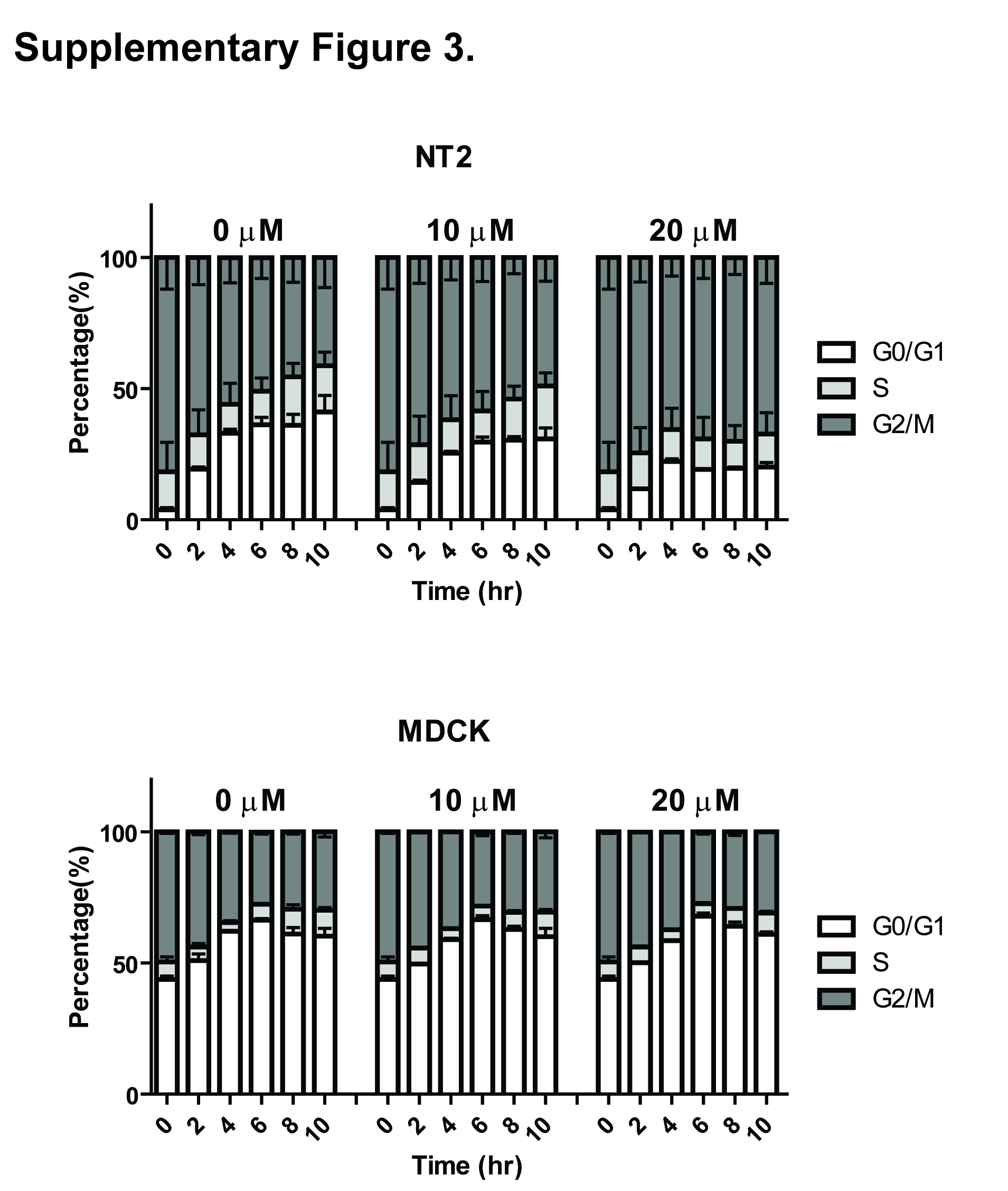

Supplement: Additional file 5 — Figure S3. Curcumin selectively blocks mitotic progression in different cell lines. NT2 and MDCK cells were arrested with thymidine/nocodazole treatment. Cells were washed and then released from mitotic block with different concentrations of curcumin for indicated time points. DNA contents were analyzed for cell cycle progression. Data are expressed as mean ± SEM of three independent experiments. [file 1471-2407-12-44-S5.TIFF]

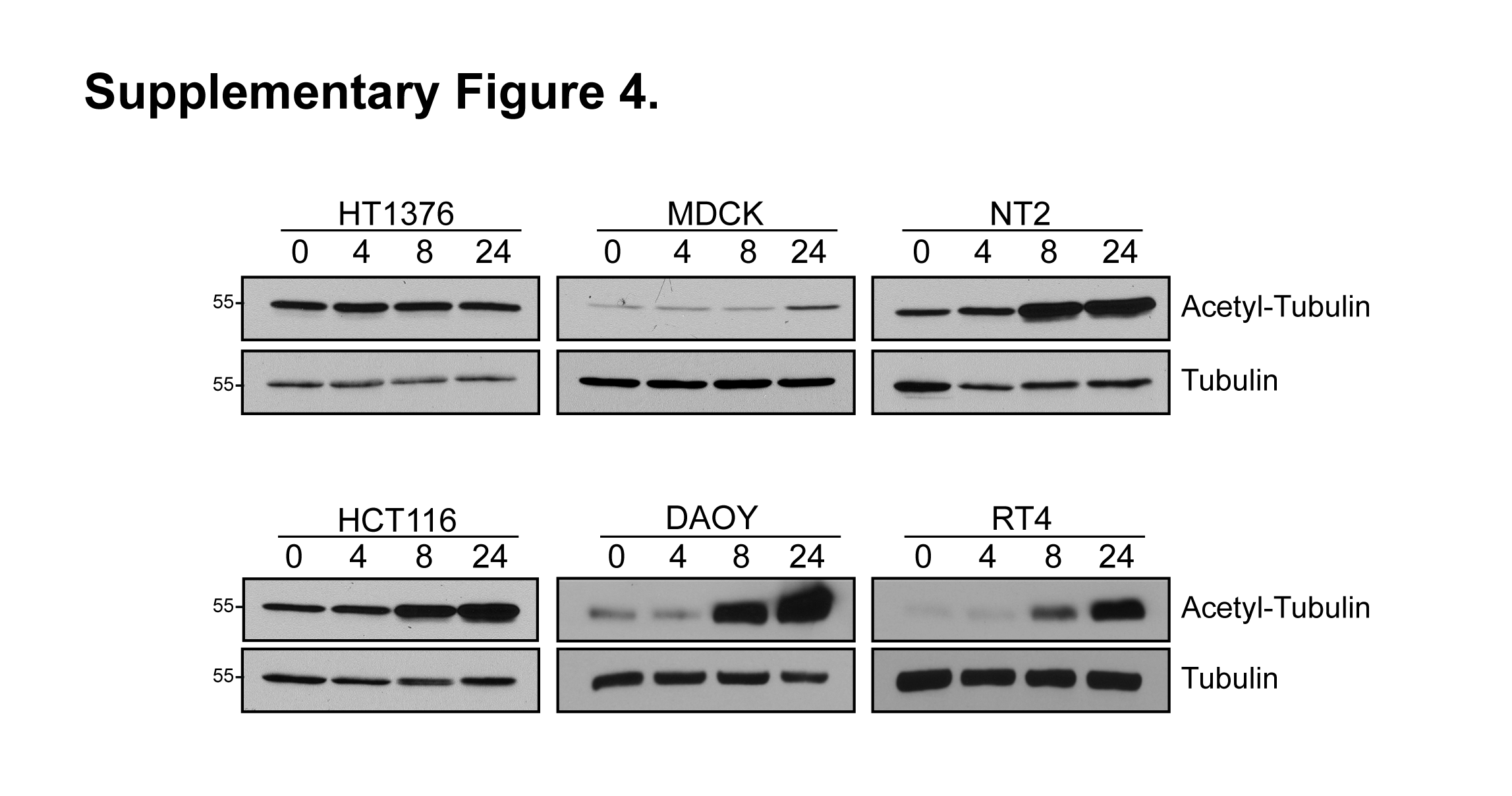

Supplement: Additional file 6 — Figure S4. Curcumin-induced acetylated tubulin accumulation. Accumulation of acetylated tubulin in six different cell lines that were incubated with 20 μM curcumin for 0, 4, 8 and 24 h. [file 1471-2407-12-44-S6.TIFF]
